# Supplementary material for: Generation of nanobodies from transgenic ‘LamaMice’ lacking an endogenous immunoglobulin repertoire
Source: Nat Commun. 2024 Jun 3;15:4728. doi: 10.1038/s41467-024-48735-x (PMC11148044; doi:10.1038/s41467-024-48735-x)
Supplement: Supplementary file 1 — Supplementary Information [file 41467_2024_48735_MOESM1_ESM.pdf]

Supplementary Information for

Generation of nanobodies from transgenic ‘LamaMice’ lacking an endogenous immunoglobulin repertoire

Thomas Eden *et al.*

Corresponding author: Friedrich Koch-Nolte, nolte@uke.de

The PDF file includes:

Supplementary Fig. 1-12

Supplementary Table 1-2

References 61-63

61. Kabat, E. A. Sequences of Proteins of Immunological Interest. U.S. Department of Health and Human Services, Public Health Service, National Institutes of Health. (1991).

62. Fumey, W. et al. Nanobodies effectively modulate the enzymatic activity of CD38 and allow specific imaging of CD38(+) tumors in mouse models in vivo. Sci. Rep. 7, 14289 (2017)

63. Kley, N., Taverniere, J., Cauwels, A. & Gerlo, S. CLEC9A BINDING AGENTS. Patent WO/2017/134301 (2017).

```
                FR1              CDR1        FR2        CDR2                    FR3
VHH1  QVQLVESGGGLVQPGGSLRLSCAASGFTLDYYAIGWFRQAPGKEREGVSCISSSDGSTYYADSVKGRFTISRDNAKNTVYLQMNSLKPEDTAVYYCAA
VHH2  QVQLVESGGGLVQAGGSLRHSCAASGLTFGSYAMGWYRQAPGKERELVAAISS-GGSTYYADSVKGRFTISRDNAKNTLYLQMNSLKPEDTAVYYCAK
VHH3  QVQLVESGGGLVQPGGSLRLSCAASGRTFSSYAMGWFRQAPGKGLEAVAAISWIGGSTYYADSVKGRFTISRDNSKNTLYLQMNSLRAEDTAVYYCAK
VHH4  QVQLVESVGGLVQDGGSLRLSCAASGRTFSRSAMRWFRQAPGKEREWVSCISSSDGSTNYADSVKARFTISRDNAKNTLYLQMNSLKPEDTAVYYCAA
VHH5  QVQLVESGGGLVQPGGSLRLSCAASGSIFSINAMGWYRQAPGKQRELVAAITS-GGSTNYADSVKGRFTISRDNAKNTVYLQMNSLKPEDTAVYYCNA
VHH6  QVQLVESGGGLVQAGGSLRLSCAASGRTFSSYAMGWFRQAPGKEREFVAAISWSGGSTYYADSVKGRFTISRDNAKNTVYLQMNSLKPEDTAVYYCAK
VH    EVQLVESGGGLVQPGGSLRLSCAASGFTFDDYAMSWVRQAPGKGLEWVSAISWNGGSTYYAESMKGRFTISRDNAKNTLYLQMNSLKSEDTAVYYCAK
```

Supplementary Fig. 1: V genes of TE02 and of TE03 LamaMice. Alignment of the deduced amino acid sequences of the VHH elements and the functional VH element. Boundaries between framework and complementarity-determining regions (FR1-FR3, CDR1, CDR2) are set according to the Kabat scheme[61]. CDR1 residues are in red, CDR2 residues in blue, four hallmark residues in FR2 in magenta. The conserved cysteine residues in FR1 and FR3 that mediate the canonical disulfide bridge are highlighted in yellow, as is the extra cysteine in VHH1 and VHH4 that can mediate a disulfide bridge to a cysteine residue in the CDR3.

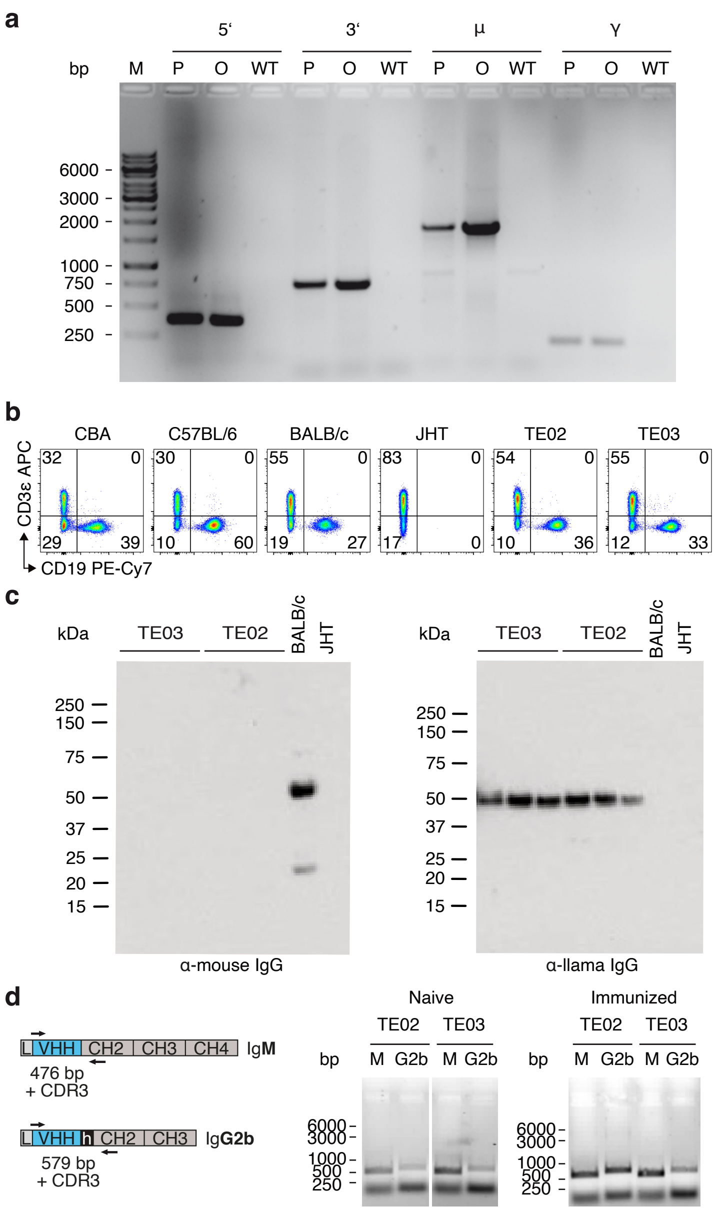

Supplementary Fig. 2: Llama IgH transgenes rescue B cell development in LamaMice. a Linearized BACs were transferred to pseudopregnant recipients via pronuclear injection. Transgene integrity and transmission to offspring was verified by PCR analysis of genomic DNA from parental mice (P) and their offspring (O). Genomic DNA from wild-type mice (WT) was used as control. PCR primers were designed to amplify diagnostic fragments from the VHH (5'), IgM (μ), IgG2b (γ) and the 3' locus control region (3'). **b** Peripheral blood cells of 9-12-week-old mice were stained with fluorochrome-conjugated mAbs against CD45, CD3 and CD19 and analyzed by flow cytometry. Gating was performed on CD45^{+} lymphocytes. **c** Immunoglobulin from serum of BALB/c wild-type mice, B cell-deficient JHT mice, and of LamaMice TE02 and TE03 was precipitated with protein A immobilized on Sepharose beads. Bound proteins were eluted from washed beads and size fractionated by SDS-PAGE. Western blots were probed with PO-conjugated antibodies specific for mouse IgG (left) or llama IgG (right). **d** Spleen cell RNA obtained from naïve and immunized TE02 and TE03 mice was PCR amplified with primers specific for llama IgM or IgG2b. PCR amplification products were analyzed by agarose gel electrophoresis.

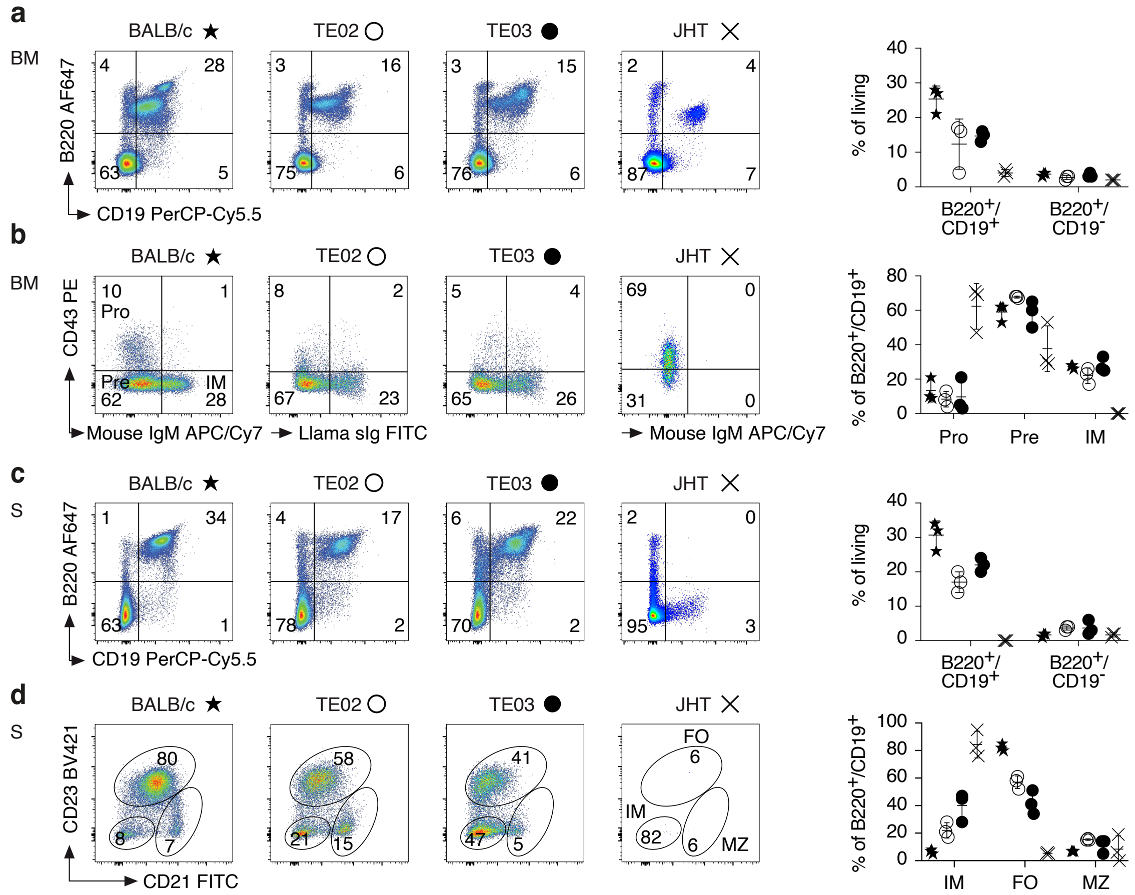

Supplementary Fig. 3: LamaMice support the development of B cells. Cells from bone marrow (BM) (**a**, **b**) and spleen (S) (**c**, **d**) of 16-27-week-old mice (n = 3 per group) were stained with fluorochrome-conjugated antibodies against the indicated markers and analyzed by flow cytometry. Gating was performed on live cells (**a**, **c**) or B220^{+}/CD19^{+} cells (**b**, **d**). Asterisks indicate samples from BALB/c mice, open circles samples from TE02 LamaMice, closed circles samples from TE03 LamaMice, crosses samples from JHT mice. Dot plots are from single representative animals. Numbers indicate the percentage of cells in the respective quadrant or gate. Bar diagrams show the corresponding results for all mice in a group. Data represent mean ± SD for n = 3 individuals.

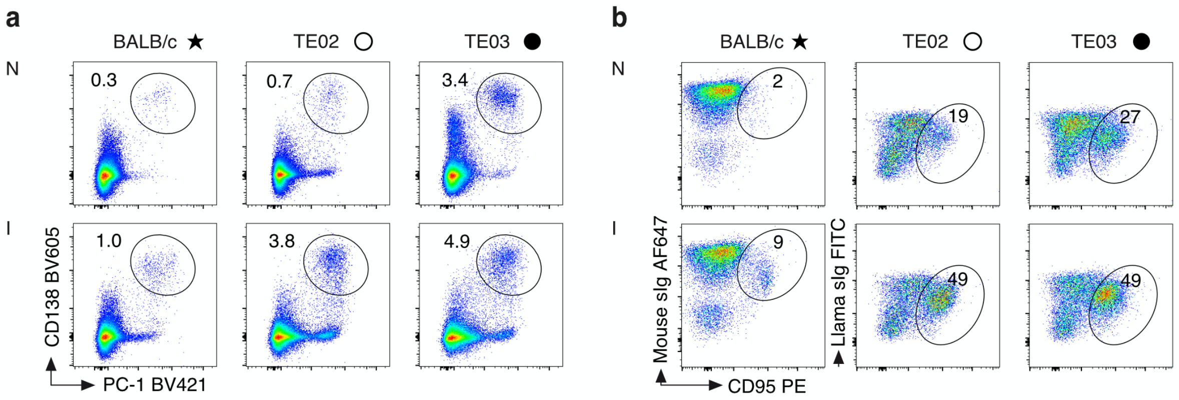

Supplementary Fig. 4: LamaMice support the development of plasma cells and germinal center B cells. **a**, **b** 12-13-week-old LamaMice (n = 3 per group) were immunized (I) with FLAG-tagged keyhole limpet hemocyanin. Age-matched naive (N) mice served as controls. Mice were sacrificed four days after the second boost and spleen cells were stained with fluorochrome-conjugated antibodies and analyzed by flow cytometry. **a** Gating was performed on NK-1.1/CD11b/CD11c triple-negative cells. Plasma cells were identified as $CD138^+$/$PC\text{-}1^+$ cells. **b** Gating was performed on $B220^+$ cells. Germinal center B cells were identified as $CD95^+$/surface Immunoglobulin $(sIg)^{int}$ cells. Asterisks indicate samples from BALB/c mice, open circles from TE02 LamaMice, closed circles from TE03 LamaMice. Dot plots are from single representative animals. Numbers indicate the percentage of cells in the gate.

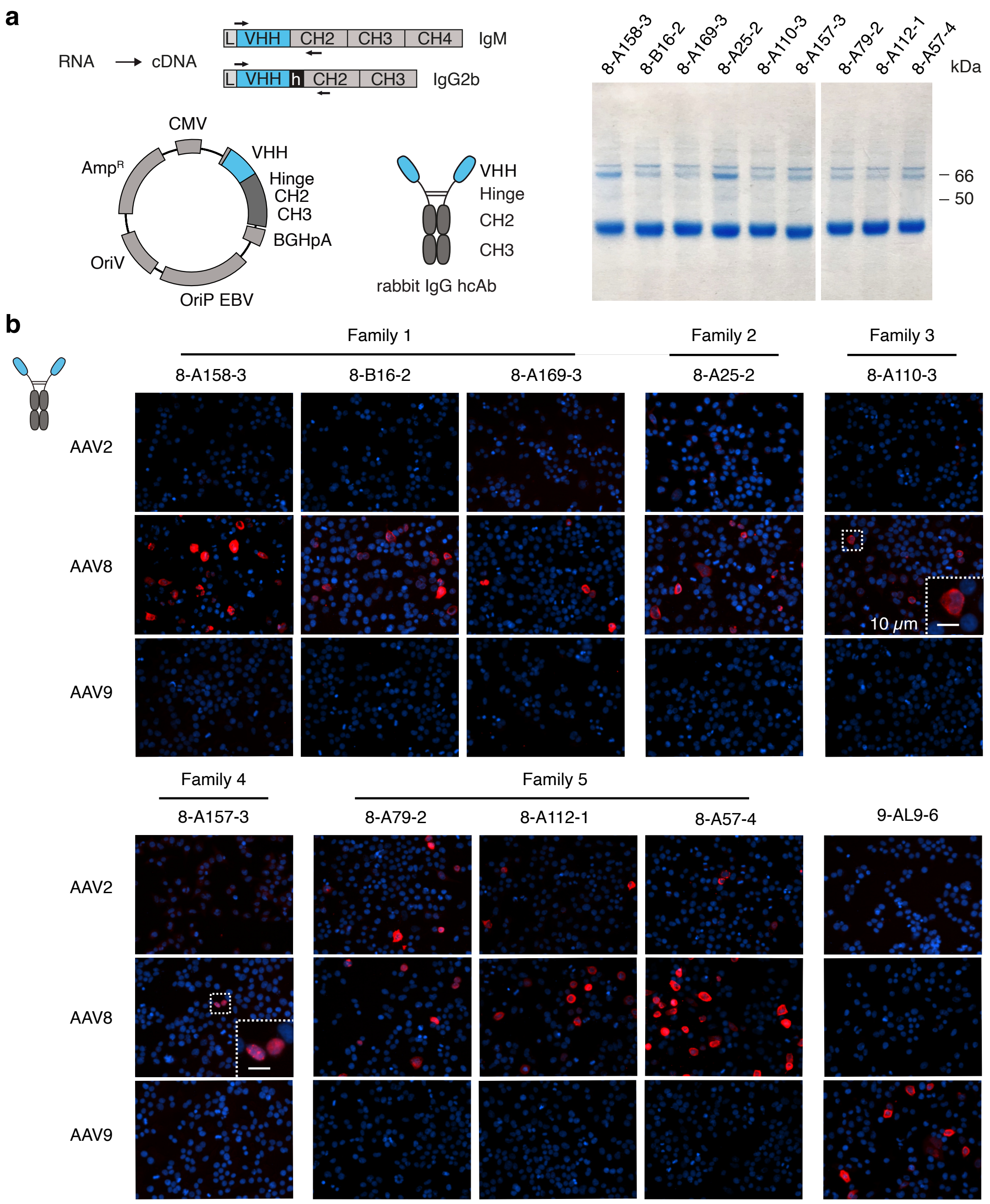

Supplementary Fig. 5: Production and characterization of AAV-specific VHH-rabbit IgG heavy chain antibodies. a The VHH-encoding region of hybridomas secreting AAV8-specific heavy chain antibodies (hcAbs) was PCR-amplified and cloned into the pCSE2.5 expression vector upstream of the hinge, CH2 and CH3 domains of rabbit IgG. VHH-rabbit IgG hcAbs were produced in transiently transfected HEK-6E cells. Six days after transfection, a 10 µl aliquot of each supernatant was analyzed for the production of hcAbs by SDS-PAGE and Coomassie staining. **b** HEK293AAV cells grown in 96-well plates were fixed in 2% PFA 48 hours after triple transfection with plasmids encoding i) adenovirus helper proteins, ii) a luciferase-encoding transgene flanked by inverted terminal repeats, and iii) the rep-cap proteins of either AAV2, AAV8, or AAV9. Wells were incubated with supernatants of HEK-6E cells. Bound antibodies were detected using PE-conjugated anti-rabbit IgG (red). Cell nuclei were counterstained using DAPI (blue). AAV9-specific 9-AL9-6-rIgG was used as control.

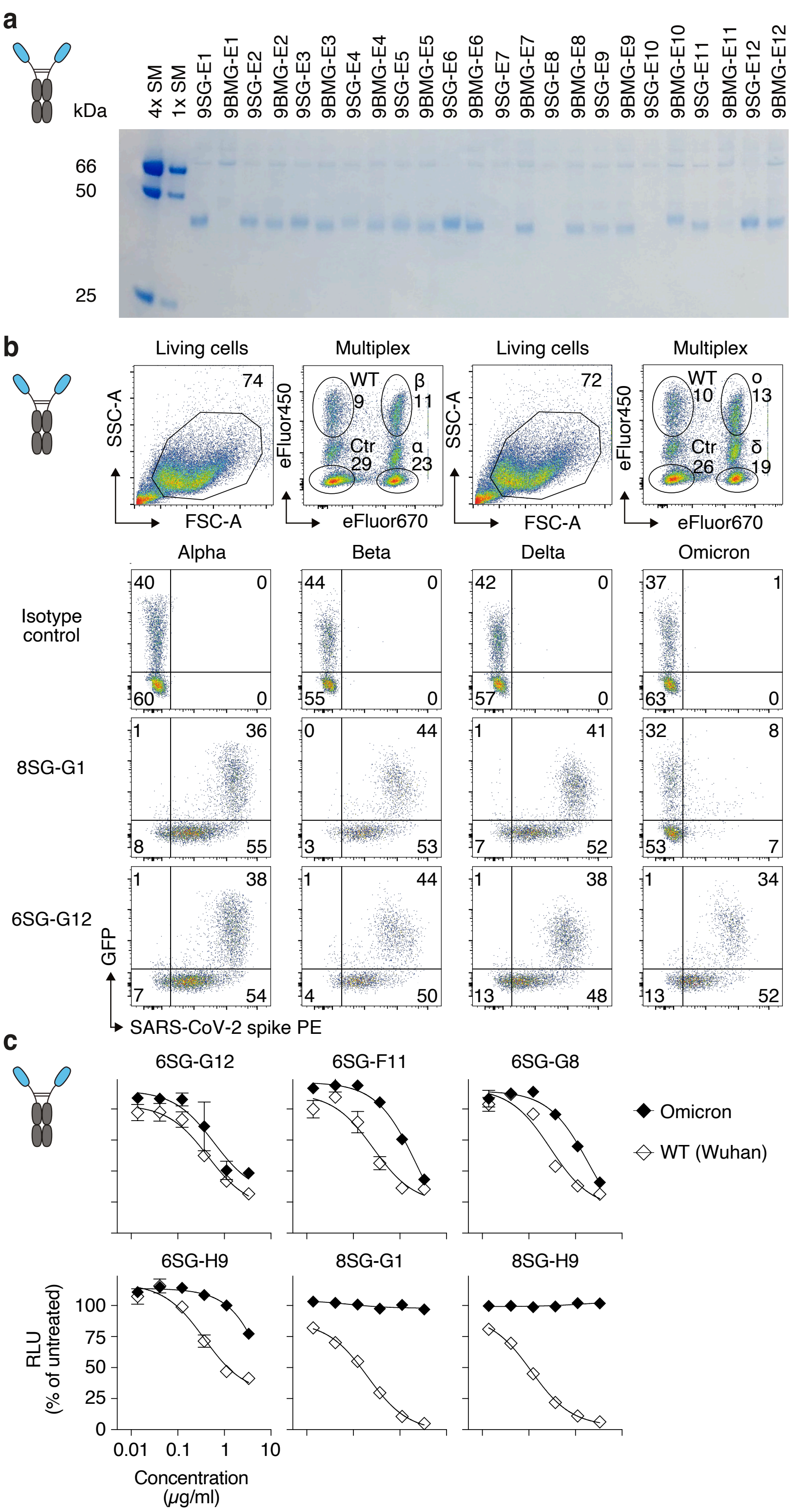

Supplementary Fig. 6: Production and characterization of RBD-specific VHH-rabbit IgG heavy chain antibodies. a The VHH-repertoire was PCR-amplified from spleen cDNA of immunized LamaMice and cloned into the pCSE2.5 expression vector upstream of the hinge, CH2 and CH3 domains of rabbit IgG. Plasmids prepared from single *E. coli* colonies propagated in 96-well plates were sequenced and transiently transfected into HEK-6E cells. Five days after transfection, a 10 µl aliquot of each supernatant was analyzed for the production

of heavy chain antibodies (hcAbs) by SDS-PAGE and Coomassie staining. **b** HEK293T cells were transiently co-transfected with expression vectors for GFP and the SARS-CoV-2 spike protein of Wuhan wild type (WT) or the indicated variants (Alpha, Beta, Delta, Omicron BA.1). Cells were harvested 24 h after transfection and analyzed by flow cytometry. To permit multiplex analyses, individual transfectants were first stained with eFluor450 and/or eFluor670, washed, and mixed before incubation with HEK-6E cell supernatants containing VHH-rabbit IgG hcAbs. Bound hcAbs were detected with PE-conjugated anti-rabbit IgG. Gating was performed to exclude cellular debris (FSC-A^{lo}/SSC-A^{lo}), and to separately analyze the subsets expressing different spike protein variants. Numbers indicate the percentage of cells in the respective gate or quadrant. Cells transfected with GFP alone served as negative control (Ctr). A *Clostridium difficile* Toxin A-specific VHH-rabbit IgG hcAb served as isotype control. **c** HEK293T cells stably overexpressing human ACE2 were incubated with luciferase-encoding lentiviral vectors pseudotyped with the spike protein of either the parental Wuhan SARS-CoV-2 (WT) or the Omicron BA.2 variant in the presence of titrated amounts of VHH-rabbit IgG hcAbs. Two days later, luciferase activity was quantified on a luminometer, 20 min after addition of luciferin. $ACE2^{+}$ HEK293T cells incubated with the respective lentiviral vector, but without hcAbs served as untreated control. Data represent mean ± SD for triplicates.

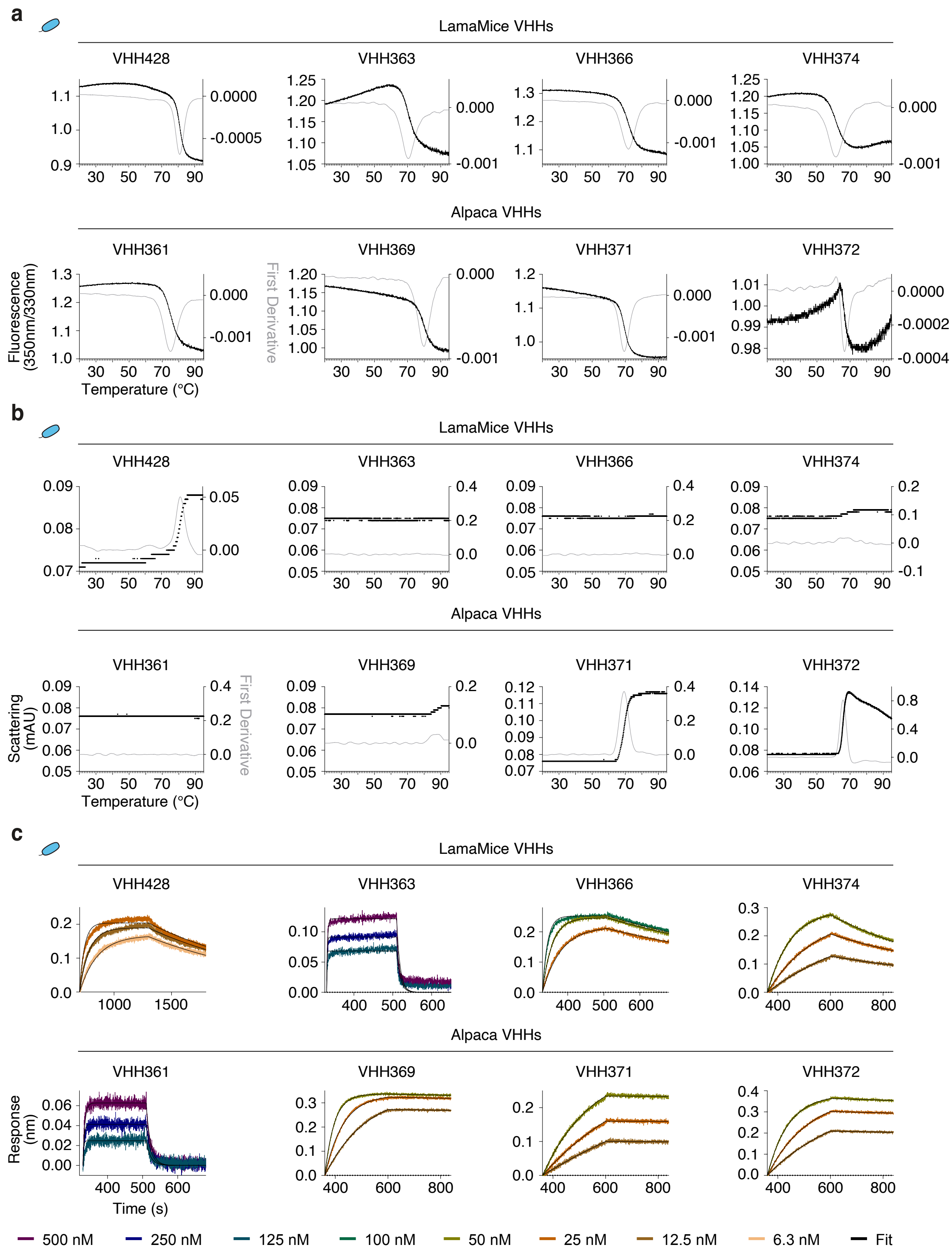

Supplementary Fig. 7: Biochemical characterisation of recombinant V_HHs from LamaMice and alpaca. Purified V_HHs from the IgE nanobody discovery campaign (see Figure 5) were analysed using nano-differential scanning fluorimetry (nanoDSF) to determine their thermal stability (**a**) and aggregation behaviour (**b**) and using biolayer interferometry (BLI) to estimate their affinity (**c**). For the BLI sensorgrams, V_HH concentrations are colour-coded according to the legend below.

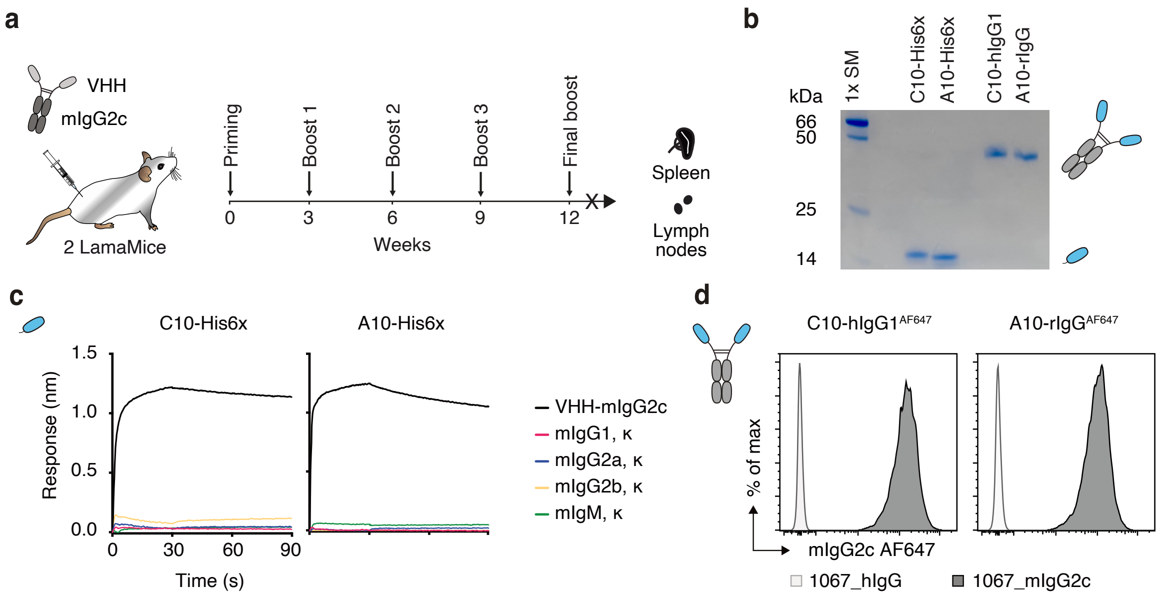

Supplementary Fig. 8: Discovery of mouse IgG2c-specific nanobodies from immunized LamaMice using direct cloning technology. a LamaMice were immunized with a purified CD38-specific VHH-mouse IgG2c heavy chain antibody (hcAb)[62]. Mouse IgG2c-specific nanobodies were identified by the direct cloning strategy. **b** VHHs of positive clones were produced as His6x-tagged nanobodies or as hcAbs with either rabbit IgG or human IgG1. Recombinant proteins were purified by affinity chromatography and analyzed by SDS-PAGE and Coomassie staining. **c** Biotinylated mouse immunoglobulins were captured on Streptavidin-coated Biosensors. Specific binding of C10 and A10 to the indicated isotypes was analyzed by biolayer interferometry. **d** Human LP-1 myeloma cells were incubated with mouse IgG2c or human IgG1 hcAbs containing the CD38-specific nanobody MU1067 (1067-mIgG2c, 1067-hIgG). Cells were washed and bound antibodies were detected with AF647-conjugated mouse IgG2c-specific hcAbs A10-rIgG or C10-hIgG1.

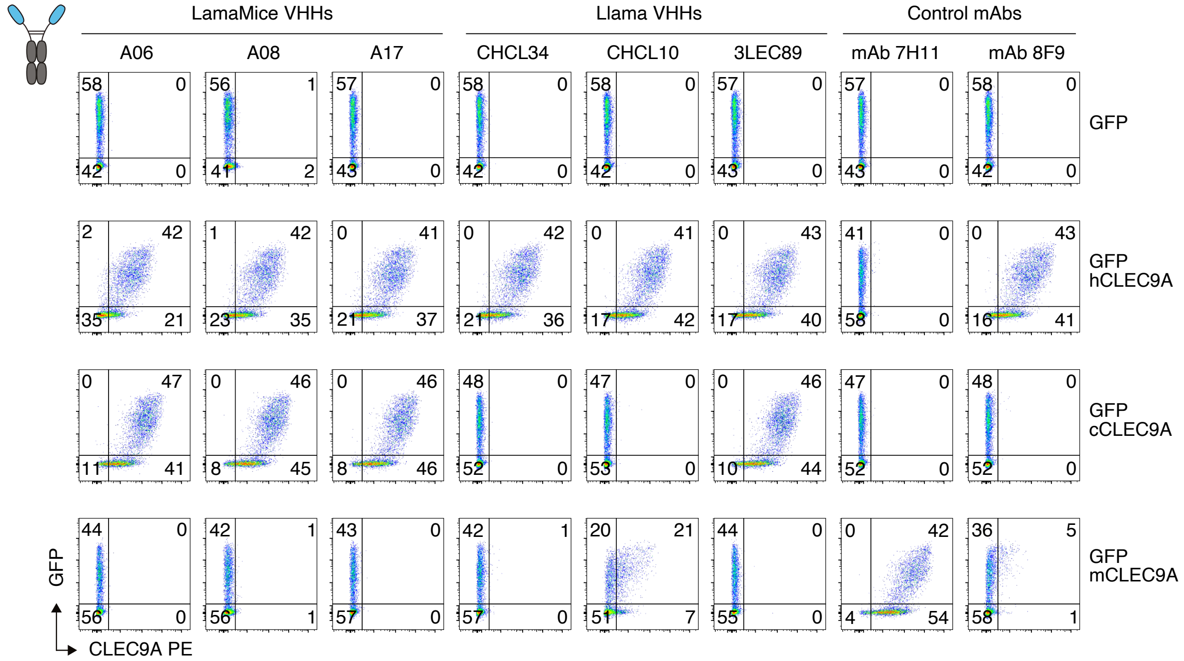

Supplementary Fig. 9: Nanobodies selected from DNA-immunized LamaMice specifically recognize human and cynomolgus CLEC9A. VHH-rabbit IgG heavy chain antibodies (hcAbs) from LamaMice (A06, A08, A17) were screened for binding to HEK293T cells transiently co-transfected with expression vectors for GFP and either human, cynomolgus or mouse CLEC9A. Parallel stainings were performed with VHH-rabbit IgG hcAbs containing nanobodies discovered from llamas immunized with recombinant human CLEC9A (R1CHCL34, R2CHCL10, 3LEC89)[49,63]. Bound hcAbs were detected with PE-conjugated anti-rabbit IgG. Commercially available, PE-conjugated mAbs against mouse (7H11) and human CLEC9A (8F9) were used as positive controls.

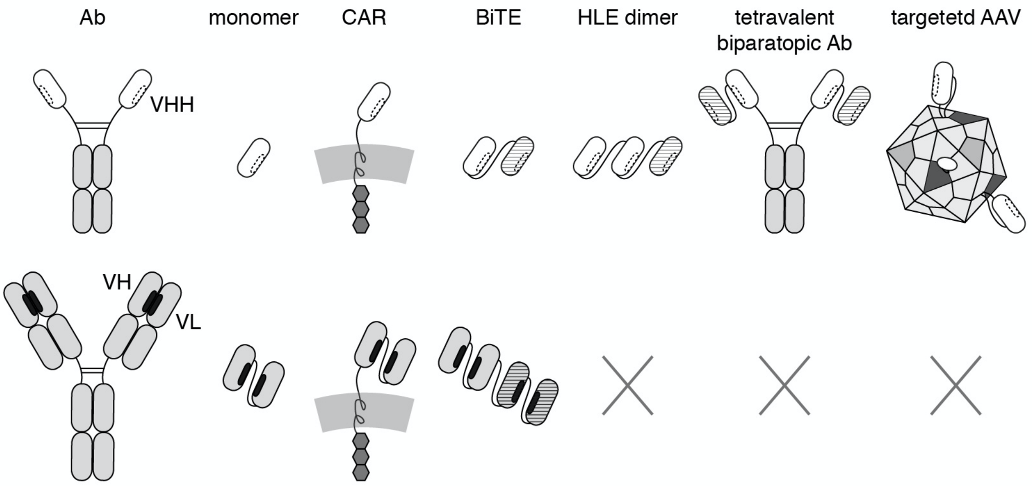

Supplementary Fig. 10: Nanobodies are readily converted into mono-, bi- and multivalent formats. Nanobodies carry a hydrophilic surface (top row, dotted patch) in the region corresponding to the hydrophobic interface of VH and VL domains (bottom row, black patches). Consequently, nanobodies can simply be fused via genetic linkers to other nanobodies and/or other protein domains. In contrast, considerable engineering efforts (X) are required to construct stable bi- and multi-specific formats with paired VH and VL domains. CAR = chimeric antigen receptor, BiTe = bispecific T cell engager, HLE dimer = half-life extended dimer, AAV = adeno-associated virus.

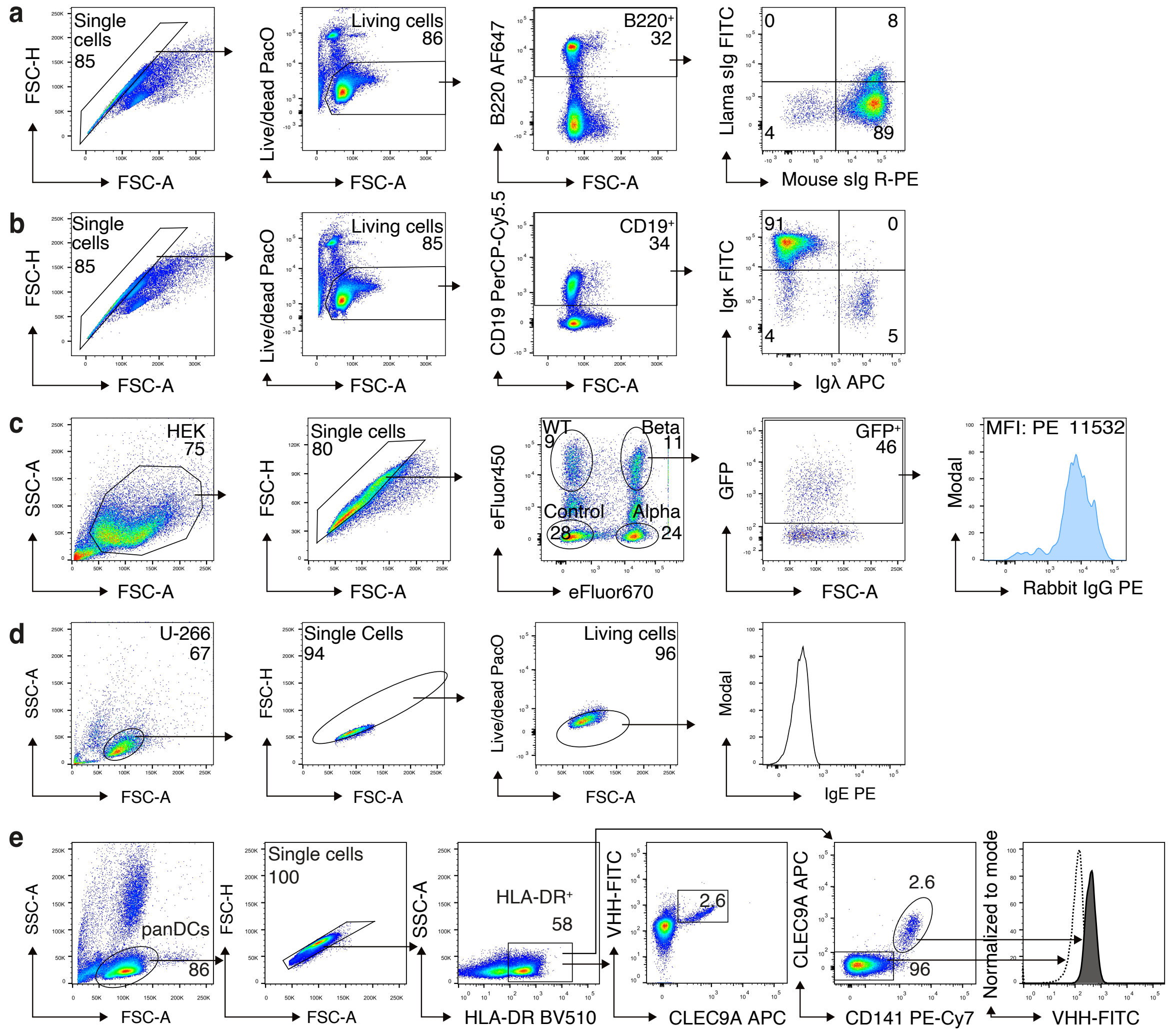

Supplementary Fig. 11: Gating strategies for flow cytometry analyses in the main figures. Shown are the gating strategies for the flow cytometry analyses in Fig. 2a (**a**), Fig. 2b (**b**), Fig. 4b (**c**), Fig. 5b (**d**), and Fig. 6c (**e**).

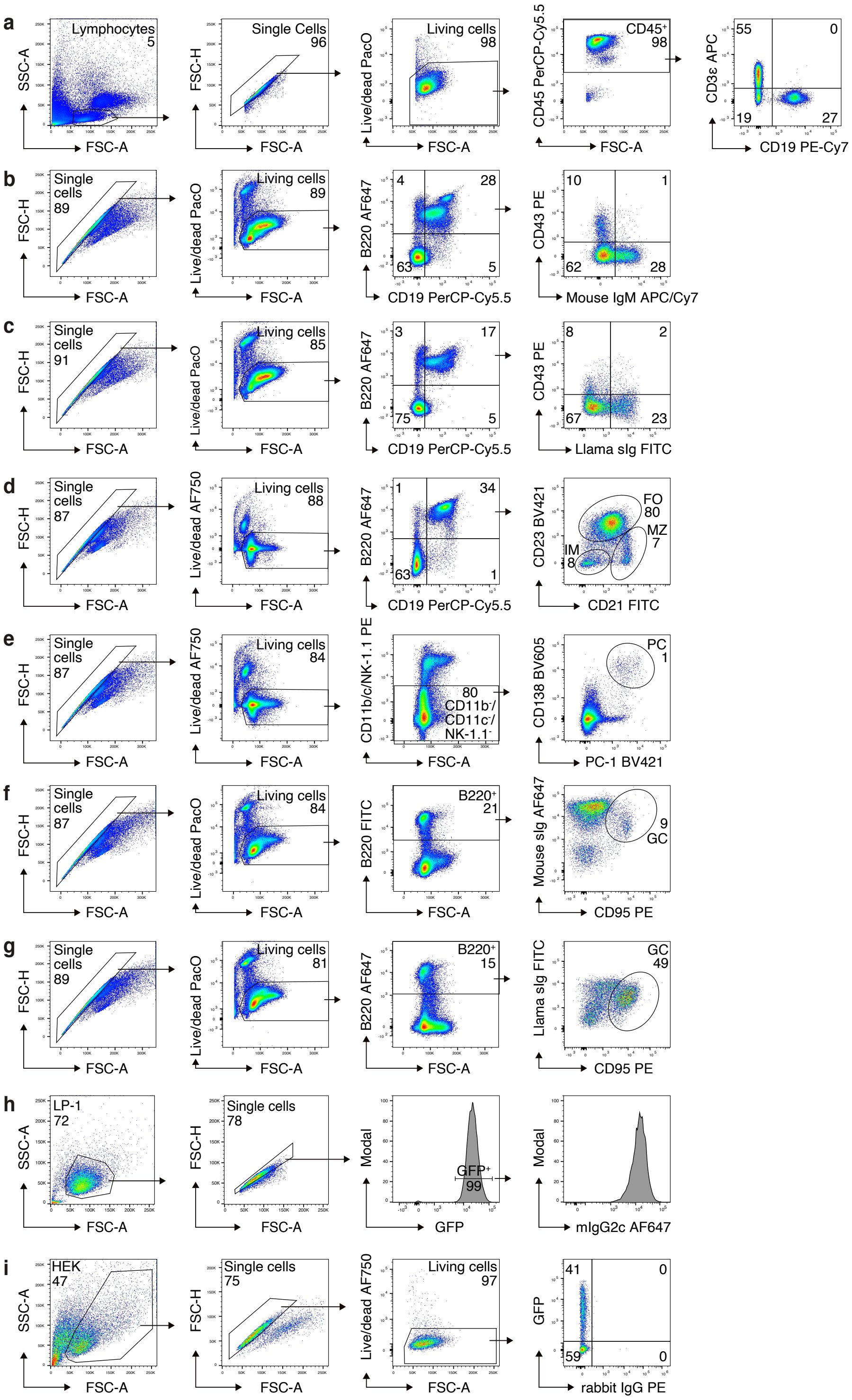

Supplementary Fig. 12: **Gating strategies for flow cytometry analyses in the supplementary figures.** Shown are the gating strategies for the flow cytometry analyses in Supplementary Fig. 2b (**a**), Supplementary Fig. 3a (BALB/c, JHT, TE02 and TE03) and Supplementary Fig. 3b (BALB/c and JHT) (**b**), Supplementary Fig. 3b (TE02 and TE03) (**c**), Supplementary Fig. 3b, c (**d**), Supplementary Fig. 4a (**e**), Supplementary Fig. 4b (BALB/c and JHT) (**f**), Supplementary Fig. 4b (TE02 and TE03) (**g**), Supplementary Fig. 8d (**h**), and Supplementary Fig. 9 (**i**).

Supplementary Table 1: Oligonucleotides used for PCR and sequencing.

name	usage	sequence (5'-3')
TE#18f	delete Cd	TCGCCACGTCCGGTCAATGCTGCCTTCCTAGCTTCCGGGGGCGGCCAGCACCT CGTTGACGTCCACATATACCTGC
TE#19r	delete Cd	GCGAAGTACTCCCAGGTGCATCCGGCCTCCGAGGTGCTGCCTGTGCTCCTGGG TGGGTCAGAAGAACTCGTCAAG
TE#20	delete Cd	GTCCGGTCAATGCTGCCT
TE#37	delete Cd	CGAGGTGCTGCCTGTGCTC
TE#39	replace loxP	GGCCTCTGTCGTTTCCTTTCTCTG
TE#40	replace loxP	CGACACCCGCCAACACCCGCTGAC
TE#41	delete CH1	GAGAGTGCTGGCATCCGCTGCATG
TE#42	delete CH1	CTGCAGGGAAAGACAGAGCGTCAG
TE#49f	VHH tg	GTGGTTGTGAGTGAGGGAATCAGGACG
TE#50r	VHH tg	TGTCTGCTGCACAGTAATAAACGGCCG
TE#47f	LCR tg	AAGGACGAGTCACAGAGGACTTCCTGG
r.pCC1	LCR tg	CTCGTATGTTGTGTGGAATTGTGAGC
TE#4f	IgM tg	CTGGCTGACACTGGGCTGACCT
TE#2r	IgM tg	AGGCGGTCAGTAGCAGGTG
TE#16	IgG tg	AATCCGTCCCTGCCCTATGCC
TE#17	IgG tg	AAGGTGGTTGGCTTGTCTGAC
TE#131f	IgG/M cDNA	GTGTCCAGGCTCAGGTGCAGCTGG
TE#85r	IgM cDNA	GCATGAAGAGTGTCACGCTGG
TE#127r	IgG cDNA	AGAGGACGTCCTTGGGTTTCGG
TE#133f	IgG/M cDNA	TCGCACATGTCTCAGGTGCAGCTGGTGGAGTCTG
TE#134r	IgM cDNA	ATGAAGAGTGTCACGCTGGGCGGCCGCCTTGGGTTCTGAGGAGACGGTGACC
TE#135r	IgG cDNA	AGGATTGGGTTGTGGTGCGGCCGCTGGTTGTGGTTTTGGTGTCTTGGGTTC
TE#136f	IgG/M NGS	ACACTCTTTCCCTACACGACGCTCTTCCGATCTGGCTCAGGTGCAGCTGGTGG AGTCTG
TE#137r	IgM NGS	TGACTGGAGTTCAGACGTGTGCTCTTCCGATCTCTTGGGTTCTGAGGAGACGG TGACC
TE#139r	IgM NGS	TGACTGGAGTTCAGACGTGTGCTCTTCCGATCTTGAGGAGACGGTGACCTGG
TE#138r	IgG NGS	TGACTGGAGTTCAGACGTGTGCTCTTCCGATCTTGGTTGTGGTTTTGGTGTCTT GGGTTC
TE#139r	IgG NGS	TGACTGGAGTTCAGACGTGTGCTCTTCCGATCTTGAGGAGACGGTGACCTGG
TE#150	IgM cDNA	GGAAGACACGTTCTTCTCGATGACC
TE#154	IgG2b cDNA	GGCCTTGGAGATGGTCTTCTCGATGG
TE#161f	PCR1	GGCTCAGGTGCAGCTGGTGGAGTCTG
TE#149r	PCR1 IgM	CACGAAACCAGGACACGGAGATCTCC
TE#153r	PCR1 IgG2b	CGCACCTCAGCGCCATCAATGTACC
TE#155f	PCR2	GGTGACATGTCTCAGGTGCAGCTGGTGGAGTCTG
TE#157r	PCR2 IgM	CACGCTGGGGGGCAGATCGGCGGCCGCTGAGGAGACGGTGACC
TE#156r	PCR2 IgG2b	TGGTTGTGGTTTTGGTGTCGCGGCCGCTGAGGAGACGGTGACC

Supplementary Table 2: Antibodies used for flow cytometry, ELISA and Western blots.

antigen	reactivity	conjugate	dilution	clone	provider
CD141	human	PE/Cy7	1:20	M80	Biolegend
CD38	human	none	1:100	MU1067	in house, UKE
CLEC9a	human	APC	1:25	8F9	Biolegend
CLEC9a	human	PE	1:200	8F9	Biolegend
CLEC9a	mouse	PE	1:200	7H11	Biolegend
HLA-DR	human	bv510	1:200	L243	Biolegend
CD11b	mouse	PE	1:200	M1/70	BD Pharmingen
CD11c	mouse	PE	1:200	N418	Biolegend
CD138	mouse	BV605	1:200	281-2	Biolegend
CD19	mouse	PerCP-Cy5.5	1:200	eBio1D3 (1D3)	Invitrogen
CD19	mouse	PE/Cy7	1:200	eBio1D3 (1D3)	Invitrogen
CD21/CD35	mouse	FITC	1:200	7G6	BD Pharmingen
CD23	mouse	BV421	1:200	B3B4	Biolegend
CD3	mouse	APC	1:200	145-2C11	Biolegend
CD43	mouse	PE	1:200	S7	BD Pharmingen
CD45R/B220	mouse	FITC	1:200	RA3-6B2	Biolegend
CD45R/B220	mouse	AF647	1:200	RA3-6B2	Biolegend
CD45	mouse	PerCP-Cy5.5	1:200	30-F11	Biolegend
CD95	mouse	PE	1:200	Jo2	BD Pharmingen
NK-1.1	mouse	PE	1:200	PK136	BD Pharmingen
PC-1	mouse	BV421	1:200	YE1/19.1	Biolegend
IgM	mouse	APC/Cy7	1:200	RMM-1	Biolegend
IgG (H+L)	llama	FITC	1:200	polyclonal	Bethyl Lab
IgG (H+L)	mouse	R-PE	1:200	polyclonal	Invitrogen
IgG (H+L)	mouse	AF647	1:200	polyclonal	Invitrogen
Ig kappa	mouse	FITC	1:200	RMK-45	Biolegend
Ig kappa	mouse	APC/Cy7	1:200	RMK-45	Biolegend
Ig lambda	mouse	APC	1:200	RML-42	Biolegend
IgG (H+L)	rabbit	R-PE	1:200	polyclonal	Jackson IR
IgG (H+L)	llama	HRP	1:2,500	polyclonal	Bethyl Labs
IgG	mouse	HRP	1:10,000	polyclonal	GE Healthcare
IgE	human	APC	1:50	MHE-18	Biolegend
