# Supplementary material for: Generation of nanobodies from transgenic ‘LamaMice’ lacking an endogenous immunoglobulin repertoire
Source: Nat Commun. 2024 Jun 3;15:4728. doi: 10.1038/s41467-024-48735-x (PMC11148044; doi:10.1038/s41467-024-48735-x)
Supplement: Supplementary file 3 — Reporting Summary [file 41467_2024_48735_MOESM3_ESM.pdf]

nature portfolio

Corresponding author(s): Friedrich Koch-Nolte

Last updated by author(s): 03/27/2024

Reporting Summary

Nature Portfolio wishes to improve the reproducibility of the work that we publish. This form provides structure for consistency and transparency in reporting. For further information on Nature Portfolio policies, see our Editorial Policies and the Editorial Policy Checklist.

Statistics

For all statistical analyses, confirm that the following items are present in the figure legend, table legend, main text, or Methods section.

n/a	Confirmed	
☐	☒	The exact sample size (n) for each experimental group/condition, given as a discrete number and unit of measurement
☐	☒	A statement on whether measurements were taken from distinct samples or whether the same sample was measured repeatedly
☐	☒	The statistical test(s) used AND whether they are one- or two-sided<br>*Only common tests should be described solely by name; describe more complex techniques in the Methods section.*
☒	☐	A description of all covariates tested
☒	☐	A description of any assumptions or corrections, such as tests of normality and adjustment for multiple comparisons
☐	☒	A full description of the statistical parameters including central tendency (e.g. means) or other basic estimates (e.g. regression coefficient) AND variation (e.g. standard deviation) or associated estimates of uncertainty (e.g. confidence intervals)
☒	☐	For null hypothesis testing, the test statistic (e.g. F, t, r) with confidence intervals, effect sizes, degrees of freedom and P value noted<br>*Give P values as exact values whenever suitable.*
☒	☐	For Bayesian analysis, information on the choice of priors and Markov chain Monte Carlo settings
☒	☐	For hierarchical and complex designs, identification of the appropriate level for tests and full reporting of outcomes
☒	☐	Estimates of effect sizes (e.g. Cohen's d, Pearson's r), indicating how they were calculated

Our web collection on statistics for biologists contains articles on many of the points above.

Software and code

Policy information about availability of computer code

Data collection	BD FACSDiva Software v9.0.2 (Becton Dickinson)<br>BD FACSSuite software (Becton Dickinson)<br>Octet BLI Discovery 12.2 (Sartorius)<br>Quantity One v.4.6.2(Bio-Rad Laboratories)<br>PR.Control v1.12.3 (NanoTemper Technologies)<br>BLItz Pro software version 1.4 (ForteBio)<br>VISION-CAPT (BIO-VISION/Abcam)<br>Wallac 1420 software version 3.00 revision 5 (PerkinElmer)<br>i-control 2.0 software (Tecan Lifesciences, CH)
Data analysis	Prism 9 version 9.4.0 and Prism 10 version 10.2.1. (GraphPad)<br>Microsoft Excel version 16.77.1 (Mircosoft Corporation)<br>FlowJo™ v10.8.1 Software (BD Life Sciences)<br>FlowJo X 10.0 7r2 (Treestar/BD Life Sciences)<br>ImageJ 1.54g (NIH)<br>Adobe Illustrator 22.0.0 (Adobe)<br>Lasergene (DNASTAR)<br>SnapGene version 7.0.2 (Dotmatics)<br>Quantity One v.4.6.2(Bio-Rad Laboratories)

Image Analyzer (Berkely Lights)
Octet BLI Analysis 12.2 (Sartorius)
PR.Control v1.12.3 (NanoTemper Technologies)
V-Quest (IMGT)
VISION-CAPT (BIO-VISION/Abcam)
Adobe Illustrator Creative Suite 5 v15.0.2 (Adobe)
Affinity Designer v1.9.3 (Serif Europe Ltd)

For manuscripts utilizing custom algorithms or software that are central to the research but not yet described in published literature, software must be made available to editors and reviewers. We strongly encourage code deposition in a community repository (e.g. GitHub). See the Nature Portfolio guidelines for submitting code & software for further information.

Data

Policy information about availability of data

All manuscripts must include a data availability statement. This statement should provide the following information, where applicable:

- Accession codes, unique identifiers, or web links for publicly available datasets
- A description of any restrictions on data availability
- For clinical datasets or third party data, please ensure that the statement adheres to our policy

All data supporting the findings of this study are included in the main article, the supplementary information, and the source data that are provided with this manuscript, including fluorescence quantifications and uncropped gels/blots. Reagents and materials described in this paper are available from the authors upon request, for which a material transfer agreement is to be executed with UKE. LaMice are available under a non-exclusive license for academic research.

Research involving human participants, their data, or biological material

Policy information about studies with human participants or human data. See also policy information about sex, gender (identity/presentation), and sexual orientation and race, ethnicity and racism.

Reporting on sex and gender	n/a
Reporting on race, ethnicity, or other socially relevant groupings	n/a
Population characteristics	n/a
Recruitment	n/a
Ethics oversight	n/a

Note that full information on the approval of the study protocol must also be provided in the manuscript.

Field-specific reporting

Please select the one below that is the best fit for your research. If you are not sure, read the appropriate sections before making your selection.

[x] Life sciences [ ] Behavioural & social sciences [ ] Ecological, evolutionary & environmental sciences

For a reference copy of the document with all sections, see nature.com/documents/nr-reporting-summary-flat.pdf

Life sciences study design

All studies must disclose on these points even when the disclosure is negative.

Sample size	As these studies were largely exploratory in character, no sample size was calculated a priori
Data exclusions	No data were excluded from the analyses.
Replication	The number of replications for each experiment is indicated in the Figure legend and/or main text. For nanobody discovery campaigns, immunization was carried out once with 2-6 animals for each antigen. The initial screening for the presence of antigen-specific antibodies was carried out once with each cell supernatant. The DNA sequences of candidate VHHs were then expressed as epitope-tagged monovalent nanobodies and/or as IgG heavy chain antibodies. Specific reactivity of recombinant nanobodies and hcAbs was verified in at least three independent experiments using ELISA, flow cytometry, immunohistochemsitry and Biolayer interferometry.
Randomization	Mice were randomized for the analysis of B-cell development (16-27 weeks of age, n = 3) and for antibody production in response to KLH-immunization (16-18 weeks of age, n = 3).
Blinding	There was no blinding to group allocations in this study since B cell numbers allow ready distinction of wildtype, IgH-deficient and LaMice.

Reporting for specific materials, systems and methods

We require information from authors about some types of materials, experimental systems and methods used in many studies. Here, indicate whether each material, system or method listed is relevant to your study. If you are not sure if a list item applies to your research, read the appropriate section before selecting a response.

Materials & experimental systems

n/a	Involved in the study
☐	☒ Antibodies
☐	☒ Eukaryotic cell lines
☒	☐ Palaeontology and archaeology
☐	☒ Animals and other organisms
☒	☐ Clinical data
☒	☐ Dual use research of concern
☒	☐ Plants

Methods

n/a	Involved in the study
☒	☐ ChIP-seq
☐	☒ Flow cytometry
☒	☐ MRI-based neuroimaging

Antibodies

Antibodies used	Primary antibodies: CD11b (PE, clone M1/70, BD, Pharmingen, Cat#553311, Lot#64266), CD11c (PE, clone N418, Biolegend, Cat#117308, Lot# B154998), CD19 (PerCP-Cy5.5, clone eBio1D3, Invitrogen, Cat#45-0193-82, Lot#2437619), CD19 (PE-Cy7, clone eBio1D3, Invitrogen, Cat#25-0193-82, Lot#2265483), CD21/CD35 (FITC, clone 7G6, BD Pharmingen, Cat#553818, Lot#75994), CD23 (BV421, clone B3B4, Biolegend, Cat#101621, Lot# B352399), CD3 (APC, clone 145-2C11, Biolegend, Cat#100312, Lot# B359445), CD43 (PE, clone S7, BD Pharmingen, Cat#553271, Lot#1340019), CD45 (PerCP-Cy5.5, clone 30-F11, Biolegend, Cat#103132, Lot# B370638), CD45R/B220 (FITC, clone RA3-6B2, Biolegend, Cat#553088, Lot#1354998), CD45R/B220 (AF647, clone RA3-6B2, Biolegend, Cat#103226, Lot# B336517), CD95 (PE, clone Jo2, BD Pharmingen, Cat#554258, Lot#M040303), CD138 (BV605, clone 281-2, Biolegend, Cat#142515, Lot# B340698), NK-1.1 (PE, clone PK136, BD Pharmingen, Cat#557391, Lot#65616), PC-1 (BV421, clone YE1/19.1, Biolegend, Cat#149207, Lot# B295307), IgM (APC/Cy7, clone RMM-1, Biolegend, Cat#406516, Lot# B201936), Human IgE (APC, clone MHE-18, Biolegend, Cat# 325507, Lot#B248125), llama IgG (H+L) (FITC, Bethyl Lab, Cat# A160-100F, Lot#24), rabbit IgG (H+L) (R-PE, Jackson IR, Cat#711-116-152, Lot#160183), mouse IgG (H+L) (AF647, Invitrogen, Cat# A-21236, Lot#2119153), mouse IgG (H+L) (PE, Invitrogen, Cat#715-116-151, Lot#135384), Ig kappa (FITC, clone RMK-45, Biolegend, Cat#409510, Lot# B211325), Ig kappa (APC-Cy7, clone RMK-45, Biolegend, Cat# 409503), CD141 (PE/Cy7, clone M80, Biolegend, Cat# 344110), CLEC9a (APC, clone 8F9, Biolegend, Cat# 353806), CLEC9a (PE, clone 8F9, Biolegend, Cat# 353804, Lot#B304540), CLEC9a (PE, clone 7H11, Biolegend, Cat# 143504, Lot#B285403), HLA-DR (BV510, clone L243, Biolegend, Cat# 307646).<br>Secondary antibodies: rabbit IgG (H+L) (R-PE, Jackson IR, Cat#711-116-152, Lot#160183), llama IgG (H+L) (HRP, Bethyl Labs, Cat# A160-100P, Lot#23), mouse IgG (HRP, GE Healthcare, Cat#NXA931)<br>The following nanobodies were derived from published literature: Nb12, Nb30 (PMID 34098567), VHH-72 (6waq), CHCL10 and CHCL34 (WO2017/134301), 3LEC89 (US2021/0024637). The following nanobodies were generated in nanobody discovery campaigns in our own labs: MU1067, VHH369, and B84#6.
Validation	All commercial antibodies were verified according to manufacturer's specifications on their corresponding websites. Nanobodies produced in our labs as monomers or heavy chain antibodies were verified by ELISA, immunofluorescence microscopy and/or flow cytometry as described for other nanobodies in this paper.

Eukaryotic cell lines

Policy information about cell lines and Sex and Gender in Research

Cell line source(s)	The following cell lines were obtained from the DSMZ-German Collection of Microorganisms and Cell Cultures GmbH: HEK293T cells (ACC 635) and LP-1 cells (femaile, ACC 41). HEK293-6E cells were kindly provided by Yves Durocher, Ottawa, Canada (PMID: 33827946). HEK293AAV cells were obtained from Cell Biolabs. HEK293T cells stably expressing the ACE2 receptor were produced in our own lab (PMID: 35643072).
Authentication	The cell lines were not authenticated.
Mycoplasma contamination	Cell lines were regularly tested for mycoplasma contamination. Only negative tested cells were used.
Commonly misidentified lines (See ICLAC register)	No commonly misidentified cell lines were used in the study.

Animals and other research organisms

Policy information about studies involving animals; ARRIVE guidelines recommended for reporting animal research, and Sex and Gender in Research

Laboratory animals	The study involved wildtype BALB/c mice, wildtype C57BL/6 mice, wildtype CBA mice, purchased from The Jackson Laboratory (Farmington, CT, USA). JHT-mice (B6.129P2-Igh-Jtm1Cgn) were kindly provided by Klaus Rajewsky, Berlin, PMID: 8513499). TE-02 and

	TE-03 LaMice were generated as detailed in the paper. Mice were housed in a specific-pathogen-free facility at temperatures of 21–24°C with 40–70% humidity on a 12h light/12h dark cycle and provided with food and water ad libitum. Mice use for the analysis of B-cell development were sacrificed at 16-27 weeks of age, mice used for KLH-immunization were sacrificed at 16-18 weeks of age. For nanobody discovery campaigns, immunizations were started when LaMice were 10-20 weeks of age.
Wild animals	No wild animals were used in this study.
Reporting on sex	Male and female mice were used in animal experiments.
Field-collected samples	No field-collected samples were used in this study.
Ethics oversight	Animal experiments were performed according to national and institutional animal care and ethical guidelines and were approved by the Veterinarian Agency of Hamburg and the local animal care committee (registration number A029/2019).

Note that full information on the approval of the study protocol must also be provided in the manuscript.

Plants

Seed stocks	n/a
Novel plant genotypes	n/a
Authentication	n/a

Flow Cytometry

Plots

Confirm that:

- [x] The axis labels state the marker and fluorochrome used (e.g. CD4-FITC).
- [x] The axis scales are clearly visible. Include numbers along axes only for bottom left plot of group (a 'group' is an analysis of identical markers).
- [x] All plots are contour plots with outliers or pseudocolor plots.
- [x] A numerical value for number of cells or percentage (with statistics) is provided.

Methodology

Sample preparation	Single cell suspensions were prepared from bone marrow, spleen, and lymph nodes of sacrificed mice. Blood samples obtained by jugular puncture from living mice. HEK cells were harvested by trypsinization and washed once with medium containing FCS. Fc-receptors were blocked with αCD16/32 and cells were stained with the indicated antibodies.
Instrument	Cells were analyzed on a BD FACSCanto II, FACSCelesta or FACSymphony using Diva (Becton Dickinson).
Software	Flow cytometry data were analyzed using FlowJo (Treestar) software. Compensation of spectral overlap was performed with the same antibodies immobilized on UltraComp eBeads (Invitrogen).
Cell population abundance	Dead cells were excluded by staining with Pacific Orange or Alexa Fluor 750 succinimidyl esters (Invitrogen).
Gating strategy	Dead cells were excluded by staining with Pacific Orange or Alexa Fluor 750 succinimidyl esters (Invitrogen). Gating was performed on cell populations expressing or lacking cell surface antigens specified in the Figure legends.

- [x] Tick this box to confirm that a figure exemplifying the gating strategy is provided in the Supplementary Information.
